# Condom Distribution in Jail to Prevent HIV Infection

**DOI:** 10.1007/s10461-012-0190-5

**Published:** 2012-05-04

**Authors:** Arleen A. Leibowitz, Nina Harawa, Mary Sylla, Christopher C. Hallstrom, Peter R. Kerndt

**Affiliations:** 1 Department of Public Policy, UCLA Luskin School of Public Affairs, Box 951656, Los Angeles, CA 90095-1656 USA; 2 UCLA Center for HIV Identification, Prevention and Treatment Services, Los Angeles, CA USA; 3 Charles R. Drew University of Medicine and Science, College of Medicine, Los Angeles, CA USA; 4 C*Change, Division of Public Health Foundation Enterprise, Inc, Los Angeles, CA USA; 5 Department of Mathematics, University of Portland, Portland, OR USA; 6 Los Angeles County Department of Public Health, Los Angeles, CA USA; 7 UCLA School of Public Health, Los Angeles, CA USA

**Keywords:** Condom, Public costs, HIV, Jail, Condón, Costos públicos, VIH, cárcel

## Abstract

To determine if a structural intervention of providing one condom a week to inmates in the Los Angeles County Men’s Central Jail MSM unit reduces HIV transmissions and net social cost, we estimated numbers of new HIV infections (1) when condoms are available; and (2) when they are not. Input data came from a 2007 survey of inmates, the literature and intervention program records. Base case estimates showed that condom distribution averted 1/4 of HIV transmissions. We predict .8 new infections monthly among 69 HIV-negative, sexually active inmates without condom distribution, but .6 new infections with condom availability. The discounted future medical costs averted due to fewer HIV transmissions exceed program costs, so condom distribution in jail reduces total costs. Cost savings were sensitive to the proportion of anal sex acts protected by condoms, thus allowing inmates more than one condom per week could potentially increase the program’s effectiveness.

## Resumen

Para determinar si la entrega de un condón por semana a reclusos de la unidad MSM de la Cárcel de Hombres del Condado de Los Ángeles reduce la transmisión de VIH y el costo social neto, se estimó el número de nuevas infecciones de VIH 1) cuando hay condones disponibles; y 2) cuando no los hay. Los datos analizados provienen de un estudio de reclusos del año 2007, de la literatura y de registros de programas de intervención. Los resultados iniciales mostraron que la distribución de condones previno ¼ de transmisiones de VIH. En base a estos resultados se puede predecir que habrán 0,8 nuevas infecciones mensuales entre 69 reclusos VIH-negativos sexualmente activos, sin distribución de preservativos, y 0,6 nuevas infecciones con preservativos disponibles. Gracias a la disminución en las transmisiones de VIH los costos médicos asociados no excedieron los costos destinados al programa, por lo que la distribución de condones en la cárcel redujo de manera efectiva los costos totales. Debido a que el ahorro en costos fue relativo al número de actos de sexo anal protegidos por condones, se puede concluir que otorgar más de un condón por semana a los reclusos podría potencialmente aumentar la eficacia del programa.

## Background

Despite several decades of prevention interventions designed to alter individual sexual or drug use behaviors, HIV infections continue to occur at an alarming rate [1]. Thus, increased attention is being directed by research and policy communities to structural level interventions, which are designed to change the context within which individuals’ decision-making occurs, for example, by removing barriers to obtaining condoms [2]. This paper examines the cost and effectiveness of one such structural intervention—a program to prevent HIV transmission by making condoms available in a jail unit for self-identified gay and transgender inmates.

Incarceration brings together people already infected or at risk for HIV infection because of their pre-incarceration behaviors such as unprotected anal sex and needle-sharing, in environments where such risky behaviors continue. Prisons and jails have been implicated as places where HIV and syphilis transmission occurs among male inmates [3–6]. In some cases health workers have used correctional institutions to access high-HIV prevalence and high-risk groups for HIV, introducing preventive measures [7, 8]. Such approaches are particularly critical given that the number of prisoners in the US has quadrupled since 1980 [9]. In 2008, there were over 1.4 million men in prisons [10] and nearly 700,000 in jails [11]. Because of the high turnover and recidivism rates associated with jails, prevention among jail inmates is also important for the larger community. Although a small number of prisons and jails in the United States provide condoms to inmates [12], there has been no analysis of the costs and effectiveness of such programs. This paper provides an analysis of a condom distribution program in the K6G protective custody unit of the Los Angeles County Men’s Central Jail, which houses self-identified gay and transgender inmates separately from other inmates.

Men who have sex with men (MSM) account for half of new HIV infections in the United States [13]. They also engage in more frequent same-sex activity (both coercive and non-coercive) in custody than men who did not have sex with men prior to incarceration [14–16]. Thus, custody units (or settings) like the K6G unit may experience particularly high rates of HIV transmission [17].

Despite the fact that the state of California classifies sexual contact in jail as a felony, officials at the Los Angeles County Men’s Jail have permitted the Center for Health Justice (CHJ), a private, non-profit organization, to distribute condoms to inmates in the segregated MSM unit for disease control purposes since 2001. The unit houses approximately 320 inmates, many of whom stay for less than 7 days (Harawa, personal communication, 2009). CHJ staff visit the unit once a week, at which time inmates line up and may receive a single condom (Harawa, personal communication, 2009). The purpose of this article is to assess the effectiveness and the net costs of the condom distribution intervention in averting HIV infections among MSM unit inmates.

## Methods

This analysis examines the cost and changes in transmission of HIV resulting from introducing condoms into a jail setting housing MSM and transgender inmates. Estimates of the amount of HIV transmission with and without a condom distribution program are made for a population of inmates with the characteristics of respondents to the 2007 survey (e.g., same share infected, same length of stay). The factors that differ between the two scenarios are the proportion of sex acts that are protected by condoms and the percent of the inmate population who engage in anal sex. This study was approved by the University of California, Los Angeles Institutional Review Board and the Charles Drew University Institutional Review Board.

### Inmate Data

Data on the characteristics of inmates and on the number of risk acts in the K6G unit when condoms are available to inmates are derived from a self-administered, computer-based survey conducted in 2007 in the MSM unit. Of the 157 randomly-selected inmates who were available for the survey (not restricted in their movements for disciplinary reasons), 111 attended an information session and were eligible for the survey because they had been incarcerated for at least 7 days, spoke Spanish or English, and were able to provide informed consent. Data are available on 101 inmates [18].

These data contain information on inmates’ reports of their sexual activity while in jail. Of the 60.4 % of respondents who had been in the MSM unit for at least 30 days, 52.6 % reported having had anal sex in jail during the prior 30 days. Those who engaged in anal sex reported an average of 9.8 encounters per month. Respondents to the 2007 survey who confirmed anal sexual activity in jail reported that they used condoms 51 % of the time, thus the 52.6 % of inmates who reported sexual activity in jail had an average of 5.0 protected acts and 4.8 unprotected acts per month.

Information on what behaviors jail inmates would have engaged in if condoms were not available is more difficult to obtain. The three analyses that have examined whether inmates’ sexual activity changed following the introduction of a condom distribution program found no evidence of a statistically significant increase in inmates’ sexual activity. In their analysis of convenience samples of inmates in the K6G MSM unit conducted in 2001, immediately prior to the initiation of the condom distribution program, and again in 2002, after condom distribution had begun, Knox and Lane [19] found no statistically significant difference between the percentage of inmates reporting anal sex in the prior period (28.5 %) and in the post period (37.3 %). Sylla et al. [7] also found no change in anal or oral sex among primarily heterosexual inmates in a San Francisco jail following introduction of a condom dispensing machine. Yap et al. [20] found no significant differences in sexual activity after a condom distribution program began in a New South Wales prison. We test the sensitivity of results to four different assumptions about the proportion of inmates who have anal sex in jail: (1) 52.6 %, equal to the rate observed in the 2007 survey; (2) 40 % of inmates; (3) 30 % of inmates; (4) 28.5 % of inmates, equal to the rate reported in 2001. For the base case, we make the conservative assumption that 40 % of inmates participate in anal sex when there is no condom distribution program, and 52.6 % participate when a condom distribution program is in place. There also were no data on the number of encounters per month among inmates who were sexually active when there is no condom distribution program. Our sensitivity analysis tests the effect of assuming half as many monthly encounters as observed in the 2007 survey.

In the 2001 survey of inmates, conducted prior to condom distribution, only 2.7 % of respondents reported that they had ever used a condom in jail [19]. Because data on the percent of sex acts protected by condoms are not available and because condoms are considered contraband and are not permitted in jail unless there is an approved distribution program in place, the modeling assumes none of the sex acts would be protected in the scenario without a condom distribution program.

The prevalence of HIV in the inmate population is an important parameter in the analysis. In the 2007 survey, 32 % of respondents reported being HIV positive [18]. In contrast, a voluntary screening program conducted in the MSM unit in 2000 and 2001 found that only 13.4 % of inmates tested positive for HIV [17]. The authors of that study note that the true HIV prevalence rate in the MSM unit is likely much higher because the screening program is voluntary and some inmates who already know their HIV status decide against testing during intake into the unit because they are already aware of their status [17]. Therefore, we use the 32 % prevalence rate in the base case for both scenarios, but test the 13.4 % rate and a 40 % rate in sensitivity analyses.

### Calculating Infections Averted by Condom Distribution

The number of infections averted is calculated as the difference between the infections predicted by a mathematical model as occurring when condom use is at the level observed in the K6G unit in 2007 and when condoms are not available to inmates.

The probability of an uninfected inmate remaining uninfected if he has unprotected anal sex with an infected inmate is (1 − *a*)^*xp*^, where *a* is the per act HIV transmission probability due to unprotected anal sex, *x* is the number of unprotected acts of anal sex over a 1-month period, and *p* is the proportion of acts with a partner who is HIV-infected. The probability that a sexual partner is HIV-infected is assumed equal to the proportion of the inmate population who are HIV-positive. Our base case uses a conservative estimate of .5 % for *a*, the transmission probability per sex act because no data were available on the percentage of acts that were receptive only or insertive only. Vittinghoff et al. [21] calculated a transmission rate of .82 % for the uninfected, receptive partner during unprotected anal sex and a rate of .06 % for the uninfected, insertive partner. Sensitivity analyses also test values of .82 and .06 %. See Table 1.

**Table 1:** Model parameters

Parameter	Base case values		Sensitivity analysis values	Source
	Without condoms	With condoms		
HIV transmission probability per unprotected anal sex act	.005	.005	.0006–.0082	Vittinghoff et al. [21]; Baggaley et al. [30]; Mastro and de Vincenzi [32]
Condom effectiveness	.85	.85	.67–.90	Pinkerton et al. [24]; Pinkerton and Abramson [25]; Weller [26]; Vittinghoff et al. [21]
% inmates HIV-positive	32.0	32.0	13.4–40.0	Harawa et al. [18]; Javanbakht et al. [17]
HIV-related lifetime medical costs, discounted to time of infection, adjusted to 2009 $.	367,121	367,121	NA	Schackman et al. [22]; US Census Bureau [23]
% inmates with anal sex	40.0	52.6	28.5–52.6 in no condoms scenario	Harawa et al. [18]; Knox and Lane [19]
Anal sex acts/inmate with sex/month	9.76	9.76	4.88–9.76	Harawa et al. [18]
% anal sex acts protected by condom	0	51.0	40–60	Harawa et al. [18]; Knox and Lane [19]

The number of uninfected inmates who become infected over a 1-month period, in the absence of condoms, is given by:

1 $$ N\left[ { 1- \left( { 1- a} \right)^{xp} } \right] $$

where *N* is the number of sexually active, uninfected inmates in the unit for at least one month.

The number of infections in this population over a 1-month period, with condom distribution, is given by:

2 $$ N\left[ { 1- \left( { 1- a\left( { 1- e} \right)} \right)^{{{\mathbf{(}}xpz )}} \left( { 1- a} \right)^{{{\mathbf{(}}xp ( 1- z{\mathbf{))}}}} } \right] $$

where *e* represents the effectiveness of condoms for preventing HIV transmission, and *z* the proportion of anal sex acts that are protected by condoms.

The number of infections averted was calculated as the difference in an individual’s probability of infection when condoms are available and when they are not multiplied by the number of sexually active, uninfected inmates who were in the unit for at least one month (*N* = 69 for the scenario with condom availability; *N* = 52.5 for the scenario with no condoms, because the proportion sexually active is assumed lower).

The mean jail stay lasted 87 days (Harawa, personal communication, 2009) for inmates who were incarcerated for at least a month, thus inmates could be exposed to HIV for multiple months. For inmates who were initially HIV-negative, we calculated the probability of remaining uninfected over a 3-month jail stay, by raising the probability of remaining uninfected in one month to the power 3.

### Calculating Net Costs

We calculate the net cost of the condom distribution program, including both intervention costs and the HIV treatment costs averted if HIV transmission is reduced. A societal perspective is employed—that is, all costs (without reference to source of funds) and benefits (no matter to whom they accrued) were considered. Since the intervention was conducted in jail, productivity losses and the value of inmate time were not included.

The lifetime cost of HIV treatment over a 32.1 year period, discounted to the time of infection, is $303,100 in 2004 $ [22]. This number was adjusted to $367,121 in 2009 $ using the medical care component of the Consumer Price Index [23]. Intervention costs (including time spent by jail staff, transportation, material, facility, and other costs) were reported by CHJ and adjusted to 2009 $ (see Table 2).

**Table 2:** Cost of intervention per month

Cost category	Units	Unit cost (US$)	Total cost (US$)
Personnel			
Intervention	17.75 h	33.77	592
Administration	2 h	33.42	67
Supervision	4 h	40.75	163
Staff transportation	80 miles	.49	39
Material	1 month	35.75	36
Facility	1 month	46.67	47
Other^a^	1 month	17.07	17
Total in 2007 $			961
Total in 2009 $			994

^a^Other costs include telephone, Internet, printing and photocopying

Net expected costs were calculated by subtracting predicted medical costs averted per month from monthly intervention costs. Averted medical costs were calculated as the product of number of infections averted and the present value of future HIV treatment costs. Sensitivity analyses tested the effect of doubling the cost of the intervention and increasing the cost tenfold.

Other model parameters, including values used for sensitivity analyses, are presented in Table 1 and were drawn from the literature [24–30].

## Results

The total cost of the intervention was $994 per month in 2009 $ (Table 2), most of which (86 %) is accounted for by personnel costs. In the base case, .8 new infections per month would be expected in the absence of a condom distribution program. With condom distribution, the incidence rate falls to .6 per month (Table 3). That is, the intervention averts .2 infections per month. More HIV infections are averted if the HIV prevalence in the inmate population is higher and at higher rates of transmission. Greater numbers of infections are averted at higher rates of condom effectiveness (90 vs. 66.7 %), if condoms are used for a larger share of the anal sex acts (60 vs. 40 %) and if a greater proportion of inmates engage in sexual activity in the absence of a condom program. Results were sensitive to assumptions about the level of sexual activity in the absence of condom availability. Condom distribution reduces HIV incidence rates if we assume equal rates of sexual activity in the scenarios with and without condom distribution. HIV transmission remains unchanged or falls when 30 or 40 % of inmates are sexually active in the absence of condom distribution. However, incidence rates are lower in the no-condom scenario if we assume that only 28.5 % of inmates are sexually active in the absence of condoms.

**Table 3:** New HIV infections with and without a condom distribution program

Parameter varied	Estimated number of new HIV infections/month		Estimated cost savings 2009 $
	No condom program	With condom program	
Base case	.82	.61	74,777
Intervention cost/month			
$1988	.82	.61	73,783
$9940	.82	.61	65,831
HIV prevalence among inmates			
13.4 %	.44	.33	39,834
40.0 %	.90	.67	82,205
HIV transmission probability/act			
.005 (Base case)	.82	.61	74,777
.0006	.10	.07	8,214
.008	1.30	.98	119,186
Condom effectiveness			
.67	.82	.71	38,697
.90	.82	.58	84,802
Share of inmates with anal sex in absence of condom program			
.285	.58	.61	−11,415
.300	.61	.61	−172
.400 (base case)	.82	.61	74,777
.526	1.07	.61	169,213
No. of acts/month among active			
9.76 (base case)	.82	.61	74,777
4.88	.41	.31	37,230
Proportion of anal sex acts protected			
.40	.82	.71	37,968
.60	.82	.53	104,933

Using base case parameters, we estimate that the probability that an individual HIV-negative inmate who is sexually active in jail becomes infected falls from 1.6 to .9 % each month when condoms are available. During an average 3 month stay, the probability falls from 4.6 % without condoms to 2.6 % if condoms are available in jail (Table 4). Over the course of an average 3 month stay in a unit without condom distribution, we predict 2.4 new HIV infections among the nearly 53 sexually active inmates who were HIV-negative at the start of their jail stay. When condoms are available, this number falls to 1.8 new infections (Table 4) among 69 inmates. Thus, .6 infections over 3 months would be averted by a condom distribution program.

**Table 4:** Number of infections and number averted by length of jail stay

	No condom program	With condom program	Infections averted
Number of uninfected, sexually active inmates in the unit	52.5	69	
Probability of infection			
After 1 month (%)	1.6	.9	
After 3 months (%)	4.6	2.6	
Number of New Infections			
After 1 month	.82	.61	.21
After 3 months	2.41	1.81	.60

Table 3 shows substantial social cost savings as a result of the reduced HIV incidence brought about by the condom distribution program. The base case indicates societal cost savings over the next 32 years of $74,777 (Table 3). These savings were not sensitive to a tenfold increase in the cost of the intervention.

## Discussion

The LA Jail condom distribution program was estimated to avert 25 % of HIV transmissions among inmates in the K6G unit, reducing the number of new infections from .8 to .6 per month. The greatest reductions occur when the underlying probability of transmission is greater (high HIV prevalence among inmates; more unprotected sexual activity in the absence of a condom distribution program, and higher HIV transmission probability per act).

An innovation of this analysis was allowing for an increase in the amount of sexual activity among jail inmates when condoms are available to them. If the model had assumed that the frequency of sexual activity remained unchanged after the introduction of condom distribution in correctional settings, as several reports in the literature suggest [7, 19, 20], our model would have predicted even greater reductions in transmission than our base case suggests. Our sensitivity analysis showed that all but one of the assumptions we tested resulted in fewer HIV transmissions. That one exception assumed that just 28.5 % of inmates would be sexually active in the absence of condoms. The fact that the 28.5 % rate was based on a 2001 convenience sample and that the literature generally shows that inmates’ sexual activity does not change following condom distribution [7, 19, 20], lead us to conclude that condom distribution reduces HIV transmission under the most plausible assumptions.

Although our model predicts substantial reductions in new HIV transmissions, some are still expected to occur. Modeling shows that the intervention could have averted a greater number of infections and been even more cost-saving, had 60 % of the sex acts been protected, rather than the reported 51 %.

The discounted lifetime cost of treating HIV is high, so even small reductions in HIV transmission result in cost savings to society. Modeling using the base case parameters indicates that condom distribution in a segregated MSM unit at the Los Angeles County Men’s Jail is a cost-saving intervention (that is, intervention costs are more than offset by future HIV/AIDS-related medical care costs avoided) when condoms are used 51 % of the time. Thus the intervention meets a higher economic threshold for acceptance than cost-effectiveness (where net intervention costs are positive but are considered reasonable, or low enough, relative to the benefits).

The cost of the intervention in the LA County jail was very modest, and the intervention remained cost-saving even if costs were ten times higher than observed. Inmates stay in jails for short periods of time and then are released back to the community, so the benefits of the reduced HIV transmission accrue to society as a whole. Our estimates of the condom distribution program’s cost saving to society would be even greater had we accounted for the reduction in future transmission of HIV by inmates who avoid infection because of condom use in jail and the benefit of preventing other sexually transmitted infections.

Although condom distribution in jails would benefit society, i.e., reduce costs in the long run, it may be difficult for the financially strapped jail systems to commit the resources necessary for this cost-saving intervention. Given that the benefits accrue to society at large, there is a compelling argument for public health funding of these initiatives.

The fact that the Los Angeles Jail restricts the number of condoms provided to one condom per week per inmate may have limited the share of inmates’ sex acts that could be protected [18]. Our cost analysis suggests that the costs of distributing additional condoms in jail would be minimal; therefore, we recommend that Los Angeles County consider increasing or eliminating its limitations on the number of condoms distributed per week in order to avert even greater numbers of HIV infections.

### Limitations

There was little information available on the amount of sexual activity that would have taken place in the absence of a condom distribution program. Although several studies support that condom distribution does not increase the amount of sexual activity in jails or prisons, we conducted several sensitivity analyses to test the effects of different assumptions about the percent of inmates with sexual activity in the absence of a condom distribution program. With one exception, these analyses as well as those assuming lower numbers of encounters per month showed the program remained cost-saving.

A limitation of the analysis, similar to many other published economic evaluations of HIV-prevention behavioral interventions, is the assumption that HIV infections avoided during the brief period when the intervention is in place represent infections prevented forever. Some of these infections are not prevented, merely delayed [31]. However, because nearly 60 % of sexually active inmates reported using condoms in the month prior to being incarcerated (Harawa, personal communication, 2009), we can expect that much of the sexual activity after release from jail would be protected. The high prevalence of HIV among inmates in the K6G unit means that the risk of infection for an HIV-negative inmate is greater while in jail than when released. Further, the high recidivism [the 2007 survey indicated that the average inmate had had 7 prior incarcerations (Harawa, personal communication, 2009)] enhances the importance of providing protection for sexual activity within jails. The public cost of HIV treatment will decline even if HIV infection is simply delayed and not permanently averted, because the present value of future treatment costs is lower if those costs are delayed to a future date.

The analysis may have understated the cost of condom distribution because the program was carried out very inexpensively in the Los Angeles jail unit by a non-profit organization, which may not be available in other settings. If the intervention were carried out by jail staff, the cost of delivering the intervention might increase. However, our sensitivity analysis showed that even if costs were higher by a factor of ten, the intervention would still be cost-saving.

The base case in our analysis used an estimate of HIV transmission probability per sex act (.005) at the low end of the range of estimated probabilities for receptive anal intercourse (.005–.03) reported by Mastro and de Vincenzi [32] to counter not being able to explicitly account for other factors that may lower transmission rates. These factors include protective actions, other than condom use that inmates may have undertaken, such as serosorting or seropositioning, on which no information was available in the 2007 survey. Serosorting has been associated with a small decrease in HIV transmission (odds ratio = .88) [33], but in a jail population, the protective effect of such measures is limited because inmates often assess whether a potential partner is HIV-positive based on unreliable information (e.g., receiving special diet meals) [18]. Further, HIV-positive respondents frequently reported sex with partners of unknown serostatus (Harawa, personal communication, 2009). Seropositioning has not been found to be significantly related to HIV transmission probability [33]. Additionally, our estimates did not account for lower transmission rates for inmates receiving ARV treatment [34]. However, relying on treatment as prevention would not provide protection against other STIs that are prevalent in the Jail, and that increase HIV transmission rates. To guard against the lack of data on other risk-reducing behaviors such as serosorting or the protective effect of ARVs on HIV transmission or taking only the insertive role, sensitivity analyses tested a low transmission rate (.06 %/act). The intervention remained cost-saving even under this assumption.

HIV prevalence was high among inmates of the K6G MSM unit and sexual activity was frequent. Thus, while condom distribution was clearly cost saving for this unit, further analyses would be needed to determine the cost-effectiveness of a condom distribution program in a jail or prison unit housing a general population of inmates where these factors may be substantially lower.

## Conclusions

This study has shown that condom distribution in the MSM unit of the Los Angeles County Jail system can reduce transmission of HIV and reduce societal costs of HIV treatment. The ability of the condom distribution programs in the Los Angeles Jail to reduce HIV transmission depends on how much risk behavior is reduced (i.e., having sufficient supplies of condoms and how consistently condoms are used). Thus, jail policies that increase access to condoms, including making them available at intake to the K6G unit, may increase the effectiveness and the cost-savings obtainable from condom distribution programs. Relaxing some of the restrictions on condom distribution in the Los Angeles Jail could make the program even more effective.
